# Comparison of Direct-to-Consumer Telemedicine Visits With Primary Care Visits

**DOI:** 10.1001/jamanetworkopen.2020.28392

**Published:** 2020-12-08

**Authors:** Tara Jain, Ateev Mehrotra

**Affiliations:** 1 Department of Medicine, Brigham & Women’s Hospital, Boston, Massachusetts; 2 Department of Health Care Policy, Brigham & Women’s Hospital, Boston, Massachusetts; 3 Department of Medicine, Beth Israel Deaconess Medical Center, Boston, Massachusetts

## Abstract

This cross-sectional study compares patients who used a direct-to-consumer telemedicine service with patients who used primary care visits in the 20 US states where the service was available.

## Introduction

A new model of direct-to-consumer (DTC) telemedicine has become increasingly popular.^1^ On a DTC company’s website or cell phone application, patients select their clinical issue and submit a medical intake form online. A clinician reviews this information and then may or may not reach out to the patient for additional information. If deemed appropriate, the clinician will send a prescription to a pharmacy or mail the medication to the patient’s home.

Over the past 2 years, DTC telemedicine companies have provided more than 1 million care visits using this model and have experienced further growth during the coronavirus disease 2019 pandemic.^2^ Despite the rapid adoption of this telemedicine option, little is known about the patients who use these companies and the visits provided, and how they compare with the US population and visits to primary care physicians (PCPs).

## Methods

This cross-sectional study included users of a DTC telemedicine service. Because data were deidentified, the study was judged by the Harvard Medical School institutional review board to be exempt from review and patient informed consent. We followed the Strengthening the Reporting of Observational Studies in Epidemiology (STROBE) reporting guideline for cross-sectional studies. A DTC telemedicine company available in 20 states provided deidentified data for all patient visits between October 1 and December 31, 2019. Based on the zip code of the residence of DTC users, we compared the patients who used the DTC telemedicine company’s services with the US population in those 20 states (eAppendix in the Supplement).

We next focused on 3 common issues managed by this DTC telemedicine company, and we compared visit characteristics with those of ambulatory PCP visits. PCP data were obtained from the National Ambulatory Medical Care Survey pooled from January 1, 2013, to December 31, 2016 (eAppendix in the Supplement). All analyses were completed using Stata, version 16 (StataCorp); differences were detected using χ^2^ and 2-sided, 2-sample proportion *t* tests. Statistical significance was defined as *P* < .05, and all comparisons were 2-sided.

## Results

Among the 35 131 DTC telemedicine visits in our sample, 25 162 (73.9%; 95% CI, 73.4%-74.4%) were from female users, and the mean (SD) user age was 36 (12) years. Compared with the overall population in these 20 states, DTC telemedicine patients were more likely to live in urban areas (85.0% vs 75.4%; *P* < .001) and areas with a higher income (32.8% vs 25.0% of the top quartile of zip code median household income; *P* < .001). Of all DTC telemedicine visits, 14.4% (95% CI, 14.0%-14.8%) were for patients living in a primary care health professional shortage area (Table 1). Primary care health professional shortage areas are designations that indicate shortages in primary care professionals based on a needs assessment conducted by state primary care offices reviewed by the Health Resources & Services Administration.

**Table 1.** Comparison of Patient Characteristics of Direct-to-Consumer Telemedicine Visits vs Population Characteristics of 20 States Where the Service Was Offered^a^

Characteristic	No. (%)^b^	
	Telemedicine visit population (n = 35 131)	Population of 20 states (n = 217 849 680)
Rurality^c^		
Urban	29 862 (85.0)	164 258 658 (75.4)
Suburban	2 277 (6.5)	21 784 968 (10.0)
Large rural	1 785 (5.0)	18 081 523 (8.3)
Small town/rural	951 (2.7)	13 724 529 (6.3)
Missing^d^	256 (0.7)	NA
Median household income quartile in states, $^e^		
Lowest	6 116 (17.4)	54 462 420 (25.0)
Second	7 821 (22.2)	54 462 420 (25.0)
Third	9 408 (26.8)	54 462 420 (25.0)
Highest	11 514 (32.8)	54 462 420 (25.0)
Missing^d^	272 (0.8)	NA
Primary care HPSA^f^	5 059 (14.4)	51 194 675 (23.5)

Abbreviations: HPSA, health professional shortage area; NA, not available.

^a^ The data include zip code data from telemedicine company visits; rurality data from the rural-urban commuting area (RUCA) data based on the 2010 US Census; income data from the American Community Survey 5-Year Estimates from 2013 through 2017; HPSA data from the US Health Resources and Services Administration updated in 2020; and state population data based on the estimated 2019 US Census. Given the comparison with the entire population of 20 US states, all differences between direct-to-consumer telemedicine visits and the population were statistically significant.

^b^ Percentages may not total 100% because of rounding.

^c^ Rurality was categorized based on a rural-urban classification system published by the Washington State Department of Health using secondary RUCA codes that emphasize population size, population density, and daily commuting pattern.

^d^ Telemedicine visits in zip codes with no equivalent zip code in comparison data.

^e^ Median household income quartiles were categorized as follows: lowest, less than $43 969; second, $43 969 to $55 595; third, $55 596 to $73 381; and highest, $73 382 or more.

^f^ Primary care HPSAs are designations that indicate shortages in primary care professionals based on a needs assessment conducted by state primary care offices reviewed by the Health Resources & Services Administration.

The 3 most common conditions for which treatment was sought via DTC telemedicine (urinary tract infection [53.0%], erectile dysfunction [21.1%], and contraception [13.0%]) accounted for 87.1% (95% CI, 86.8%-87.5%) of total DTC telemedicine visits and 2.3% (95% CI, 2.3%-2.3%) of PCP visits. After limiting the analysis to these 3 conditions, and compared with PCP visits, patients who used DTC telemedicine services were more likely to be between the ages of 18 and 44 years (74.1% vs 29.4%; *P* < .001) and were less likely to self-report a comorbid condition (14.2% vs 63.8%; *P* < .001) (Table 2). Most DTC telemedicine visits (63.3%; 95% CI, 62.8%-63.8%) took place outside normal PCP business hours (9 am to 5 pm on weekdays or weekends) and rarely resulted in referrals to an emergency department or urgent care facility (0.8% of visits; 95% CI, 0.7%-0.9%).

**Table 2.** Comparison of Direct-to-Consumer Telemedicine Visits With Primary Care Physician Visits for Urinary Tract Infection, Erectile Dysfunction, and Contraception^a^

Characteristic	No. (%)	
	Telemedicine visits (n = 30 627)	Primary care physician visits (n = 29 600 000)^b^
Sex		
Male	7 442 (24.3)	10 004 800 (33.8)
Female	23 185 (75.7)	19 595 200 (66.2)
Age, y^c^		
18-44	22 705 (74.1)	8 702 400 (29.4)
45-65	7 601 (24.8)	9 531 200 (32.2)
>65	320 (1.0)	10 330 400 (34.9)
Day of week		
Weekday	23 001 (75.1)	29 067 200 (98.2)
Weekend	7 626 (24.9)	NA^d^
Source of payment for visit		
Out of pocket	30 627 (100.0)^e^	NA^d^
Insurance (all types)	NA	26 196 000 (88.5)
Other^f^	NA	2 812 000 (9.5)
Presence of comorbid conditions	4 349 (14.2)	18 884 800 (63.8)

Abbreviation: NA, not available.

^a^ The data source was the authors’ analysis of electronic medical record data from a telemedicine company and survey data from the National Ambulatory Medical Care Survey (NAMCS). Differences in each category are significant (*P* < .001).

^b^ Number of estimated total visits to primary care physicians from January 1, 2013, through December 31, 2016, based on sampling weights for these conditions. This number is based on a sample of 793 records in the data.

^c^ One user of a telemedicine visit was younger than 18 years.

^d^ Individual estimates based on fewer than 30 sample records were not reported owing to low reliability.

^e^ The direct-to-consumer telemedicine company does not accept insurance for patient visit. Insurance may be used for prescription through pharmacy.

^f^ Other includes the NAMCS payment categories labeled blank, unknown, other, or no charge/charity.

## Discussion

This study found that patients who use DTC telemedicine services were younger, tended to live in wealthier urban communities, and typically accessed care outside normal business hours. Among the services offered, only 3 issues (urinary tract infection, erectile dysfunction, contraception) accounted for 87.1% of visits compared with 2.3% of PCP visits.

Direct-to-consumer telemedicine companies advertise their potential to improve health care access. Access has many dimensions, including accommodation, acceptability, availability, and affordability.^3^ Our findings suggest the model may address accommodation barriers, such as inconvenient hours and appointment systems. Younger, more technologically savvy patients may consider online care as simply more convenient. Given the conditions managed, the model may also be attractive to who are uncomfortable receiving in-person care for sexual issues (an acceptability barrier). In contrast, DTC telemedicine does not appear to preferentially attract those with clinician availability or affordability barriers. This may be result from various factors, such as limited awareness about such services, lack of access to broadband services, or out-of-pocket visit costs.^4^

Our study has some limitations. The data are limited to a single company, influenced by the set of services offered by the company and advertising for these services; DTC telemedicine companies that offer video visits may have different patterns. Certain demographic characteristics may be overrepresented compared with the US population if patients using telemedicine had more than 1 visit in the 3-month study period. Prior assessments of telecontraception have shown care equivalent or superior in quality to PCP visits.^5,6^ However, further research is necessary to determine whether this model delivers appropriate care across a range of other conditions and its impact on routine preventive health screening.

## Notes

Supplement. **eAppendix.** Additional Methods
