# Supplementary material for: Comparison of Direct-to-Consumer Telemedicine Visits With Primary Care Visits
Source: JAMA Netw Open. 2020 Dec 8;3(12):e2028392. doi: 10.1001/jamanetworkopen.2020.28392 (PMC7724555; doi:10.1001/jamanetworkopen.2020.28392)
Supplement: Supplement. — eAppendix. Additional Methods [file jamanetwopen-e2028392-s001.pdf]

Supplementary Online Content

Jain T, Mehrotra A. Comparison of direct-to-consumer telemedicine visits with primary care visits. *JAMA Netw Open.* 2020;3(12):e2028392. doi:10.1001/jamanetworkopen.2020.28392

eAppendix. Additional Methods

This supplementary material has been provided to give readers additional information about their work.

© 2020 Jain T et al. *JAMA Network Open*.

eAppendix. Additional Methods

A DTC telemedicine company provided deidentified data for a total of 35,131 patient visits between October and December 2019. States where the company was available included AK, CA, FL, GA, IL, KY, MI, MN, MO, MT, NC, NE, NY, OH, PA, SC, TN, TX, WA, and WY.

In our first analysis, we compared the characteristics of people in zip codes with telemedicine visits to publicly available United States population data classified by zip code and restricted to the 20 states included in the study.

We categorized level of rurality as urban, suburban, large rural or small town/rural by using the secondary Rural-Urban Commuting Area (RUCA) codes based on the 2010 decennial census classified by zip code using data updated by the Economic Research Service. Categories were determined based on Guidelines for using Rural-Urban Classification Systems published by the Washington State Department of Health (Scheme 1 created with an emphasis on population size, population density, and daily commuting pattern can be accessed at https://www.doh.wa.gov/Portals/1/Documents/1500/RUCAGuide.pdf).

Median household income data by zip code was based on the 2013-2017 American Community Survey accessed through the US Census Bureau (data: Median Household Income in the Past 12 Months). We obtained primary care Health Professional Shortage Area data by zip code from the Health Resources & Services Administration, updated in 2020, and compared to state-specific HPSA data published in the HRSA FY 2020 second quarter summary. Primary care HPSAs are designations that indicate shortages in primary care professionals based on a needs assessment conducted by State Primary Care Offices reviewed by the Health Resources & Services Administration. Data from the 2019 United States census were used to determine the population of 20 states studied.

In our second analysis, we compared additional telemedicine visit data including reason for visit, sex, age, day of appointment, source of payment, and comorbidities to ambulatory visit data obtained from the National Ambulatory Medical Care Survey (NAMCS). The NAMCS is based on patient record forms completed by physicians across the United States. Data were pooled from the most recent years available (2013-2016) in order to provide stable estimates. Patient visits were weighted and extrapolated to produce annual national estimates of ambulatory visits, defined by the svyset command in Stata. We limited NAMCS data analysis to PCP visits defined by general/family practice and internal medicine, and further by the top three reasons for DTC telemedicine visits including urinary tract infection, erectile dysfunction, and contraception, which totaled 793 sample records representative of a total 29.6 million visits.

Because data were deidentified, the study was judged by the Harvard Medical School institutional review board to be exempt from review and informed consent.

© 2020 Jain T et al. *JAMA Network Open*.
